# Association between SNP12 in estrogen receptor α gene and hypospadias: a systematic review and meta-analysis

**DOI:** 10.1186/s40064-016-2288-0

**Published:** 2016-05-11

**Authors:** Changkai Deng, Rong Dai, Xuliang Li, Feng Liu

**Affiliations:** Department of Pediatric Surgery, Chengdu Women’s and Children’s Central Hospital, Chengdu, 610091 China; Department of Urology Surgery, Children’s Hospital of Chongqing Medical University, Ministry of Education Key Laboratory of Child Development and Disorder, Key Laboratory of Pediatrics in Chongqing (CSTC2009CA5002), Chongqing International Science and Technology Cooperation Center for Child Development and Disorders, Chongqing, 400014 China; Chengdu Center for Disease Control and Prevention, No. 4, Longxiang RD, Wuhou District, Chengdu, 610041 China

**Keywords:** *ESR1*, SNP12, Hypospadias, Meta-analysis, Trail sequential analysis

## Abstract

To investigate the association between single nucleotide polymorphism 12 in estrogen receptor α gene and hypospadias, four databases (PubMed, Web of Science, Embase and Cochrane Library) were electronically searched by 2nd November 2015. Finally, four studies were included for our meta-analysis, involving 1379 cases and 1648 controls. A quality assessment was performed using the Newcastle–Ottawa Scale of case–control study. Meta-analysis and publication bias measuring were all done by Stata 12.0. No significant publication bias (P_Begg_ = 0.296, P_Egger_ = 0.161) was found. Overall, there was statistically significant association for recessive genetic model (AA vs. GA + GG: OR 3.45, 95 % CI [1.89, 6.30], P = 0.038). Moreover, the positive result was confirmed using trial sequential analysis even only three original studies. For allele model, there was also statistically significant association (allele A vs. G: OR 1.43, 95 % CI [1.23, 1.67], P = 0.034). Meanwhile, A allele as a risk factor turned out to be true positive by trial sequential analysis. In a word, this meta-analysis suggested that the single nucleotide polymorphism 12 definitely increase the risk of hypospadias.

## Background

Hypospadias is the second most common congenital malformations of the male external genitalia among male newborns, in which the urinary opening is located along the ventral side of the penis. The prevalence of hypospadias varies widely between countries and populations, ranging from 3.9 to 43.2 cases per 10,000 births (Kurahashi et al. 2004; Nassar et al. 2007). Hypospadias occurs most frequently in whites, less frequently in blacks and rates are lowest among Asians and Hispanics (Carmichael et al. 2003, 2007; Yang et al. 2004; Nelson et al. 2005; Porter et al. 2005).

The etiology of hypospadias is unclear in most of the cases, which is probably regarded as a complex disorder with several genes, hormonal environment and their interaction (Kalfa et al. 2010; van der Zanden et al. 2012). In the past decades, the hypospadias susceptibility genes research mainly concentrated in both the gene of male gender differentiation and androgen receptors. On the one hand, studies focused on genes associated with sex differentiation involving *SRY*, *SOX9*, *DAX1* and *WT1* (Diller et al. 1998; Domenice et al. 1998; Kaefer et al. 1999). On the other hand, studies concentrated on luteinizing hormone (LH) receptor gene, testosterone synthase genes, 5α reductase gene and androgen receptor gene (Silver and Russell 1999; Latronico 2000).

In recent years, estrogen receptor (ER) related gene with hypospadias is a new research hotspot, especially estrogen receptor α gene (*ESR1*). *ESR1* is on chromosome 6, which expresses in most cells of the male urethra (Dietrich et al. 2004). Previous studies have investigated the association between SNP 12 (rs6932902) and hypospadias. One study (van der Zanden et al. 2010) suggested that SNP 12 borderline associated with hypospadias. But another three studies (Watanabe et al. 2007; Tang et al. 2011; Choudhry et al. 2015) identified that *ESR1* associated with significantly increased risk of hypospadias. However, the results of the original studies were inconsistent and the sample sizes were small. Therefore, in order to overcome the limitation of individual studies, we performed this meta-analysis to provide a more precise and comprehensive estimation.

## Methods

### Data source

Four databases (PubMed, Web of Science, Embase and Cochrane Library) were electronically searched to retrieve studies on the associations between SNP12 in *ESR1* and hypospadias by 2nd November 2015. Searching terms were: (‘estrogen receptor’ OR ‘*ESR1*’ OR ‘*ESR1* single-nucleotide polymorphism12’ OR ‘rs6932902’) AND (‘male genital abnormalities’ OR ‘hypospadias’). In additional, we checked the reference lists of retrieved reviews to identify more pertinent studies.

### Inclusion criteria

Titles and abstracts of all relevant papers were reviewed firstly. Then, full-texts were reviewed as a second screening. The studies were considered eligible if they met all of the following criteria: (1) the study explored the relationship between *ESR1* polymorphism and hypospadias; (2) the *ESR1* polymorphism was tested in the study; (3) the papers identified the odds ratio (OR) and the 95 % confidence interval (CI) or other information that can help to infer the target data; (4) the study design was a case–control study; (5) when multiple publications reported on the same or overlapping data, the most recent article or the article based on the largest study population was selected; (6) the publication language was English.

### Exclusion criteria

Studies met any of the following exclusion criteria were excluded: (1) researches based on animals or cells rather than general population; (2) reviews, editorials, meeting abstracts, and commentaries; (3) articles with no target data or no relevant outcomes.

### Data extraction and quality assessment

Two reviewers (Changkai Deng and Rong Dai) independently searched the literatures and selected the papers; then, they extracted the relevant data in accordance with the pre-formed data extraction form. Disagreements were solved by discussion and a third party (Xuliang Li and Feng Liu) was involved when necessary. The basic information was extracted from each article: first author, year of publication, country where study was conducted, ethnicity of subjects, sample size of case and control groups, demographic characteristics of case and control groups (age), study design, method of genotyping assay, deviation from Hardy–Weinberg Equilibrium (HWE).

Quality assessment was conducted for each article by using the Newcastle–Ottawa Scale of case–control study. The quality of the studies was evaluated by examining three items: selection of case and controls, comparability of groups and ascertainment of exposure. Studies were graded on an ordinal star scoring scale with higher scores representing studies of higher quality. A study can be awarded a maximum of one star for each numbered item within the selection and exposure categories and a maximum of two stars can be given for the comparability of the two groups. The quality of each study was graded as high quality (7–9) and low quality (1–4). For the present studies, Changkai Deng and Rong Dai scored each included study independently.

### Statistical analyses

ORs with their 95 % CIs for alleles and genotypes were used to assess the strength of association between the SNP12 in *ESR1* and hypospadias. Heterogeneity among included studies was checked by Chi square-based *Q* test and *I*^2^ test. If the data showed low heterogeneity (P > 0.05, I^2^ < 50 %), Mantel–Haenszel fix effect model was used, otherwise DerSimonian–Laird random effect model was used. The pooled ORs were performed for the allele contrast (A vs. G) and recessive genetic model (AA vs. GA + GG). HWE was tested for included studies using an online HWE calculation tool and publication bias was assessed by Egger’s linear regression test. Data were analyzed using Stata 12.0. A *P* value of 0.05 for any test or model was considered to be statistically significant unless otherwise specified.

### Publication bias

Potential publication bias was assessed by the Begg rank correlation test and Egger linear regression test using the software of Stata 12.0.

### Trail sequential analysis (TSA)

TSA can adjust the threshold for statistical significance according to the quantified strength of evidence and the impact of multiplicity. A meta-analysis may result in type I errors and type II errors if data are sparse or if there is repeated testing for significance when new trials are added (Brok et al. 2008; Borm and Donders 2009; Wetterslev et al. 2009). To minimize the risk of type I errors, TSA combines conventional meta-analysis methodology with meta-analytic sample size considerations and methods for repeated significance testing on accumulating data in trials. The trial sequential analysis was performed to maintain an overall 5 % risk of a type I error and 20 % of the type II error. We applied a constant continuity correction of 0.5 in the no event trial.

## Results

### Literature search

**Fig. 1:**
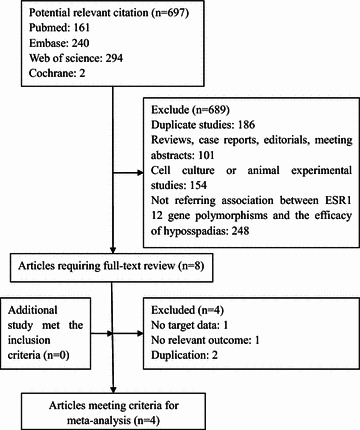
Flow chart of literature selection. Six hundred and ninety-seven publications were excluded after screening of titles and abstracts. Four eligible articles were excluded from eight relevant articles included in our full-text selection. The reasons for exclusion were as follow: one study was lack of target data; one study was no relevant outcome and two studies reported on the same or overlapping data. Finally, we identified four eligible articles

We initially identified 697 potentially eligible studies. Most of them were excluded after the screening of titles and abstracts. The main excluded reason was duplication, reviews, non population-based studies or irrelevant to SNP12 in *ESR1* and hypospadias. After assessing the full-text of eight potentially relevant articles, we identified four eligible articles. The main reasons for exclusion were as follow: one study was lack of target data; one study was no relevant outcome and two studies reported on the same or overlapping data (Fig. 1).

### Study characteristics

Four studies with 1379 cases and 1648 controls were included in the analysis. The basic characteristics of included studies are presented in Table 1. All studies were published between 2007 and 2015. The included studies were conducted in China, Dutch, Japan and the USA, respectively. Moreover, the studies involve Caucasian, Chinese, Japanese, Hispanic and White. All the included studies were case–control studies.

**Table 1:** Characteristic of the included studies

First author	Year	Setting	Ethnicity	Sample size (T/C)	Age (years) (T/C)	Design	Genotyping assay	Score	HWE	
									*χ* ^2^	*P*
L. F. van der Zanden	2010	Dutch	Caucasian	617/596	NA	Case–control	*Taq*Man	6	0.000	0.982
M. Watanabe	2007	Japan	Japanese	43/135	0–27/4–16 and 24–50	Case–control	PCR	8	1.520	0.218
K. F. Tang	2011	China	Chinese	72/40	2–19/1–15	Case–control	PCR	7	0.500	0.480
S. Choudhry	2015	USA	Hispanic and White	647/877	NA	Case–control	NA	8	NA	NA

*NA* not available, *HWE* Hardy–Weinberg equilibrium, *PCR* polymerase chain reaction, *T* case group, *C* control group

### Quantitative data synthesis

**Fig. 2:**
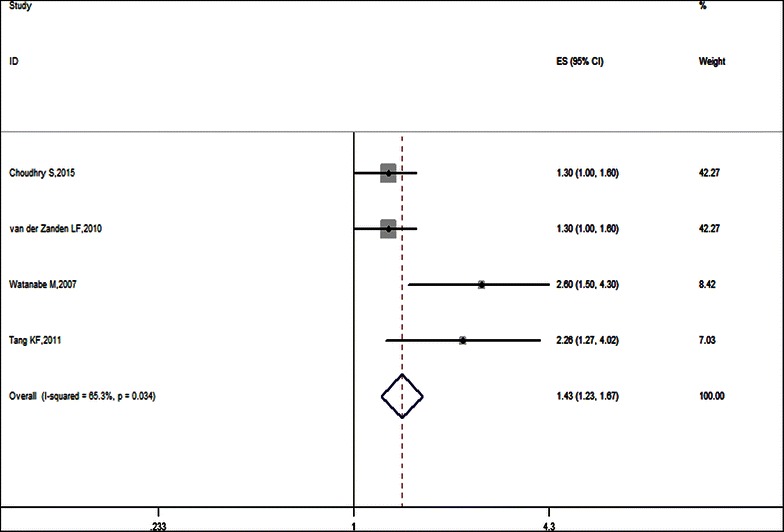
Forest plot of SNP12 and hypospadias risk in allele model. Studies are plotted according to the first author’s name and publication year. *Horizontal lines* represent 95 % CI. *Each square* represents the OR point estimate and its size is proportional to the weight of the study. The *diamond* (and *broken line*) represents the overall summary estimate, with confidence interval given by its width. The *unbroken vertical line* is at the null value (OR 1). *CI* confidence interval, *OR* odds ratio

**Fig. 3:**
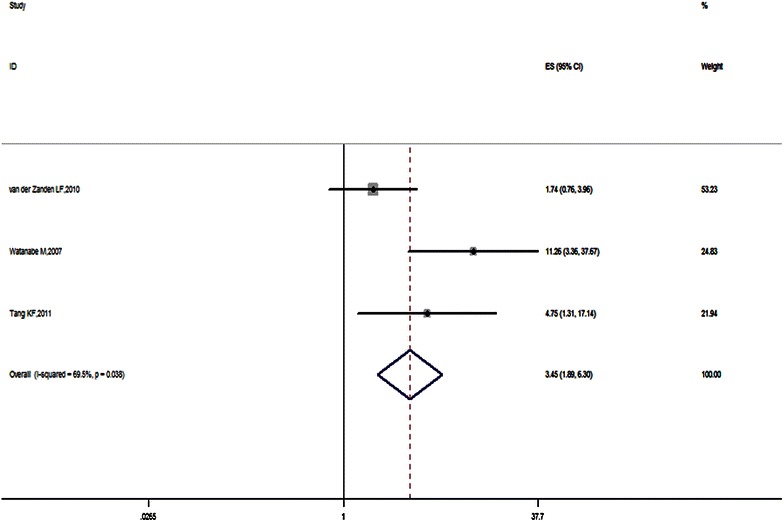
Forest plot of SNP12 and hypospadias risk in recessive genetic model. Studies are plotted according to the first author’s name and publication year. *Horizontal lines* represent 95 % CI. *Each square* represents the OR point estimate and its size is proportional to the weight of the study. The *diamond* (and *broken line*) represents the overall summary estimate, with confidence interval given by its width. The *unbroken vertical line* is at the null value (OR 1). *CI* confidence interval, *OR* odds ratio

The main results of this meta-analysis and the heterogeneity tests are presented in Figs. 2 and 3. Overall, a significant association between SNP12 and hypospadias was found for the allele contrast (allele A vs. G: OR 1.43, 95 % CI [1.23, 1.67], P = 0.034) and recessive genetic model (AA vs. GA + GG: OR 3.45, 95 % CI [1.89, 6.30], P = 0.038).

### Publication bias

The results of the Begg’s rank correlation test and Egger’s linear regression test supported the conclusion of no significant publication bias (P_Begg_ = 0.296, P_Egger_ = 0.161).

### Trail sequential analysis (TSA)

**Fig. 4:**
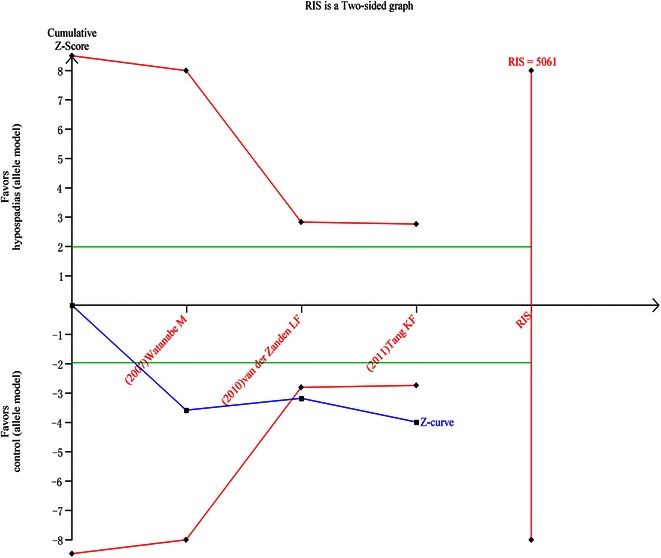
Result of TSA for allele model. Required information size was 5061 patients (α = 5 %, β = 20 %). The *blue* cumulative Z-curve crosses the inward sloping *red* trial sequential monitoring boundary for benefit during the second trial but does not reach the required information size. The *horizontal green lines* illustrate the conventional level of statistical significance (two-sided α = 0.05), which was intersected after the first trial. *Square symbol* z-score for single study, *diamond symbol* trial sequential monitoring boundary for benefit score for single study

**Fig. 5:**
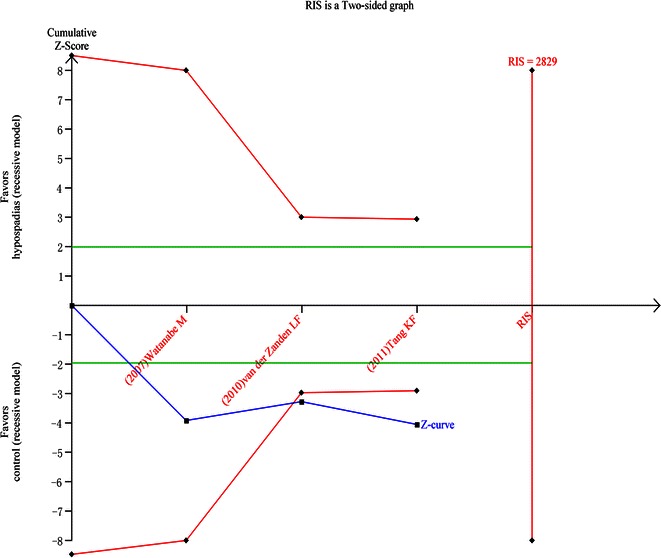
Result of TSA for recessive gene model. Required information size was 2829 patients (α = 5 %, β = 20 %). The *blue* cumulative Z-curve crosses the inward sloping *red* trial sequential monitoring boundary for benefit during the second trial but does not reach the required information size. The *horizontal green lines* illustrate the conventional level of statistical significance (two-sided α = 0.05), which was intersected after the first trial. *Square symbol* z-score for single study, *diamond symbol* trial sequential monitoring boundary for benefit score for single study

We calculated the required information size to 5061 patients for allel gene model and found that A allele as a risk factor turned out to be positive (Fig. 4) As for recessive genetic model, we calculated the required information size to 2829 patients and the positive result was confirmed (Fig. 5).

## Discussion

Hypospadias is congenital malformations of the male external genitalia. The etiology of hypospadias is a complex disorder involving several genes, hormonal environment and their interaction. It is claimed that disruption in androgen and estrogen balance, made by endocrine disrupting chemicals (EDCs) during fetal development may result in formation of abnormal male urethra (Toppari and Skakkebaek 1998; Baskin et al. 2001; Steinhardt 2004; Vidaeff and Sever 2005). Consistent with this theory, association of estrogen including fetal exposure to diethylstilbestrol, a synthetic estrogenic compound, has been associated with higher occurrence of hypospadias in humans (Swan 2000; Kalfa et al. 2011). Estrogen is taken up by a cell via *ESR1* which is ligand-activated transcription factor composed of several domains important for hormone binding. *ESR1* has been reported to be functionally active in human fetal penile smooth muscle cells (Crescioli et al. 2003; Dietrich et al. 2004). In recent years, some studies have investigated the relationship between the SNP12 in *ESR1* and hypospadias, but there exit inconsistency among all results. Thus, in this meta-analysis, our goal was to determine whether the SNP12 in *ERS1* gene associated with hypospadias.

We included fours studies with 1379 cases and 1648 controls in this Meta-analysis finally. Overall, our meta-analysis has increased the power to detect a potential association and provides more reliable estimates. Furthermore, publication bias is not present in this study. The pooled OR of recessive genetic model (AA vs. GA + GG) is 3.45 (1.89, 6.30) and allele contrast (A vs. G) is 1.43 (1.23, 1.67). The results indicate that there is significant association between SNP12 in *ESR1* and hypospadias. It suggests that AA in recessive genetic model and allele A in allele model both are risk factors to hypospadias. However some authors have suggested that conventional meta-analysis should not be trusted without further evaluation, as cumulative meta-analyses of trials are at risk of producing random errors because of sparse data and repetitive testing of accumulating data (Wetterslev et al. 2008; Higgins et al. 2011). Therefore, we challenged the meta-analyses with the application of TSA that widens the CIs in case the data are too sparse to draw firm conclusions. With this strict approach the increased risk of AA in recessive genetic model remained statistically significant, even though there were merely three original studies. Meanwhile, the result of TSA indicated that A allele as a risk factor turned out to be true positive. In conclusion, our study provides evidence that SNP12 in *ESR1* influence the risk of hypospadias.

### Limitations

In this meta-analysis, there are some limitations which may influence the findings. Firstly, we focused on individual SNP. Polymorphisms occur at low frequencies in many ethnic groups and are unlikely to explain the variation in response when considered alone. Secondly, our meta-analysis has not taken the possibility of SNP–SNP, gene–gene or gene–environment interactions into consideration.

## Conclusion

This meta-analysis suggested that the single nucleotide polymorphism 12 definitely increase the risk of hypospadias.
